# Uptake of Manganese from the Manganese-Lysine Complex in Primary Chicken Intestinal Epithelial Cells

**DOI:** 10.3390/ani9080559

**Published:** 2019-08-15

**Authors:** Shiping Bai, Keying Zhang, Xuemei Ding, Jianping Wang, Qiufeng Zeng, Huanwei Peng, Jie Bai, Yue Xuan, Zuowei Su, Bin Wu

**Affiliations:** 1 Institute of Animal Nutrition, Sichuan Agricultural University, Huimin Road 211#, Wenjiang District, Chengdu 611130, Sichuan, China; 2 Key Laboratory of Animal Disease-resistant Nutrition, Ministry of Education, Sichuan Agricultural University, Huimin Road 211#, Wenjiang District, Chengdu 611130, Sichuan, China; 3 Chinese Chelota Group, Liangshui Industrial Estate, Jinyu District, Guanghan 618300, Sichuan, China

**Keywords:** manganese lysine complex, manganese uptake, intestinal epithelial cells, chicken

## Abstract

**Simple Summary:**

Manganese (Mn) supplementation is especially necessary to avian species because the absorption of dietary Mn is relatively inefficient in birds. Recently, there has been increasing interest in the use of organic Mn to replace inorganic Mn as dietary Mn supplements in poultry. This study compared the uptake of Mn from Mn-lysine complex (MnLys) and MnSO_4_ in the primary chicken intestinal epithelial cells when the Fe, *N*-ethylmaleimide (a transport system y^+^ inhibitor), or cycloheximide (a transport system b^0,+^ activator) added in the culture medium. The results revealed that the uptake of Mn from the MnLys complex not only might be transported through the ionized Mn^2+^ pathway, but also appeared to be transported through the transport systems y^+^ and b^0,+^ in the intestine of chickens.

**Abstract:**

Organic manganese (Mn) sources can replace inorganic Mn as dietary Mn supplements in poultry. To compare the uptake of Mn from the Mn-lysine complex (MnLys) and MnSO_4_, we first established the primary chicken intestinal epithelial cells (IECs) model and used it to determine Mn uptake. The MnLys increased the uptake of Mn compared to MnSO_4_. The uptake of Mn decreased in the IECs with Fe addition in the medium regardless of the Mn sources. The MnLys decreased the Mn^2+^ efflux transporter ferroportin 1 (FPN1) mRNA level but did not influence the Mn^2+^ influx transporter divalent metal transporter 1 (DMT1) mRNA expression when compared to MnSO_4_. The results above indicated that the increase of Mn accumulation for MnLys at least partly was due to the decrease of Mn efflux by reduced FPN1 expression. The addition of *N*-ethylmaleimide, an L-lysine transport system y^+^ inhibitor, decreased the uptake of Mn from MnLys but did not affect the uptake of Mn from MnSO_4_. The cycloheximide, as an L-lysine transport system b^0,+^ activator, increased the uptake of Mn from MnLys, whereas they did not influence the uptake of Mn from MnSO_4_. The MnLys increased the system y^+^ members cationic amino acid transporter (CAT) 1 and CAT2, and system b^0,+^ components rBAT and b^0,+^AT mRNA expression when compared to MnSO_4_. These results suggested that the uptake of MnLys complex might be transported by CAT1/2 and system b^0,+^, which was different from the ionized Mn^2+^ uptake pathway. In conclusion, the uptake of Mn from MnLys complex not only might be uptake through the ionized Mn^2+^ pathway, but also appeared to be transported through the CAT1/2 and system b^0,+^ in primary chicken IECs.

## 1. Introduction

Manganese (Mn) is necessary for normal homeostatic processes controlling reproduction, the formation of connective tissue and bone, carbohydrate, lipid metabolism, and brain function [1]. The utilization of Mn has been an increasing concern because of its low efficiency of absorption from foods to animals [2,3]. Several organic Mn forms, including metal amino acid complexes, have been developed as dietary Mn supplements in poultry. Much research demonstrated that the Mn-amino acid complex was more bioavailable than inorganic Mn sulfate in ligated chicken duodenal loop [4,5] and Caco-2 cell models [6]. However, several studies reported no differences in the Mn bioavailability between organic and inorganic Mn sources [7,8]. The differences in the ligands of organic Mn forms and the evaluation criterion might partly explain the discrepancies in relative bioavailability estimates among the research reports. Therefore, it is necessary to compare the uptake of Mn from manganese-lysine complex (MnLys) or Mn sulfate (MnSO_4_). 

Intestinal epithelial cell (IEC) lines used in nutrient absorption experiments mostly were derived from colonic adenocarcinoma (e.g., Caco-2) [6,9]. However, these cell lines may not be suitable models for nutrient transport analysis, as they had undergone changes and selection to facilitate long-term growth in vitro. The primary IECs are cells taken directly from living duodenal epithelial tissue, especially the crypts of Lieberkühn located at the base of the intestinal villi, and established for growth in vitro [10]. These cells have undergone very few population doublings and are more representative of the main functional component of the duodenal epithelium in comparison to continuous cell lines [11]. The primary IECs were suitable for investigating the absorption of trace mineral elements [12,13,14]. Over the past decade, there have been several advances in our understanding of the molecular mechanisms involved in ionized Mn absorption. In particular, two Mn transport proteins have been well characterized using in vitro and rodent models: the cellular Mn importer, divalent metal transporter 1 (DMT1) [4,15], and cellular Mn exporter, ferroportin 1 (FPN1) [16,17]. There were two assumptions about the mechanism of mineral amino acid complex absorption in the small intestine of animals [18]. One was that the ionized metal from the metal amino acid complex could be absorbed as an ionized metal [4,19]. According to this assumption, the uptake of Mn from the manganese-lysine complex (MnLys) was mediated by the ionized Mn absorption pathway. Another was that the mineral chelated with amino acid was absorbed intact through an amino acid transporter [20,21,22]. According to this theory, two L-lysine transport systems, b^0,+^ and y^+^ [23,24,25], might mediate the uptake of Mn from MnLys in the enterocyte. Much research demonstrated that N-ethylmaleimide (NEM) was an inhibitor of system y^+^/cationic amino acid transporters (CAT) in human erythrocytes and rat cardiac ventricular myocytes [26,27]. The cycloheximide (CHX) was an activator of system b^0,+^ in Caco-2 cells [28,29,30]. The objective of this study was to investigate the influences of the addition of Fe, NEM, or CHX in culture medium on the uptake of Mn from MnLys or Mn sulfate in primary chicken IECs.

## 2. Materials and Methods

All experimental procedures were approved by the Institutional Animal Care and Use Committee (IACUC) of Sichuan Agricultural University for Biological and Biomedical models (approval No. SAU-13-147).

### 2.1. Materials and Antibodies

Dulbecco’s modified Eagle’s medium (DMEM)/F-12 and insulin-transferrin-selenium-X mix were from Thermo Fisher Scientific Inc., Madison, WI, USA. Fetal bovine serum, dispase II, collagenase XI, penicillin, sorbitol, streptomycin, heparin, epidermal growth factor, and Tween-20 were of the highest grade from Sigma, St. Louis, MO, USA. The 0.2 μm filters used to sterilize media were from Millipore, Bedford, MA, USA. The protein assay reagent was from Bio-Rad Lab, Richmond, CA, USA. The mouse anti-chicken cytokeratin-18 antibody was from Thermo Fisher Scientific Inc., Waltham, MA, USA, and the secondary fluorescein isothiocyanate (FITC)-conjugated goat anti-mouse IgG antibody and bisbenzimide (Hoechst 33342) were from Molecular Probes, Eugene, OR, USA. The mouse anti-chicken vimentin antibody was from RayBiotech Life Inc., Norcross, GA, USA, and the secondary goat anti-mouse IgG antibody conjugated to fluorescein 3H-indolenine trimeethine cyanine (Cy3) was from Boster, Pleasanton, CA, USA. The manganese-lysine complex (MnLys) was the compound with one atom Mn chelated by two molecules of L-lysine and contained 13.8% Mn (Chengdu Amino Acids Chelation Biotechnology, Chengdu, China). All other reagents were obtained from Sigma unless otherwise specified.

### 2.2. Intestinal Epithelial Cells Isolation and Culture

Fertile Cobb broiler eggs were purchased from a commercial hatchery (Sichuan Yuguan Agricultural Co. Ltd., Suining, China) and incubated on a wire mesh in a semi-automated incubator. Chicken embryos from these eggs at days 16–18 were euthanized by cervical dislocation. The duodenal segments were removed, and intestinal epithelial cells (IEC) were isolated using a previous method with some modification [31]. After gently rinsing with warm PBS, the duodenal loop segments were split lengthwise and sliced using a sterile scalpel blade. Slices were forced through a stainless steel mesh (55 mm diameter 100-micron opening). The resulting fragments were collected, washed, and digested in the DMEM/F12 containing 25 mmol/L glucose, 0.4 mol/L sodium bicarbonate, 0.1 mg/mL dispase II, and 300 U/mL collagenase XI for 25 min at 37 °C. The digested solution was centrifuged three times at 300 × *g* for 3 min in DMEM/F12 containing 2% sorbitol. Supernatant fluids containing non-dispersed IEC, non-epithelial cells, and debris were discarded. The remaining pellets were re-suspended in DMEM/F-12 containing 25 mmol/L glucose, 4 mmol/L glutamine, 0.4 mol/L sodium bicarbonate, 1% nonessential amino acids, 100 U/mL penicillin, 0.1 mg/mL streptomycin, 1% insulin-transferrin-selenium-X, 2.5% foetal bovine serum (FBS), 50 mg/mL heparin, and 20 ng/mL epidermal growth factor. The cells were seeded in the six-well cluster Falcon tissue culture dishes coated with rat-tail collagen (Thermo Fisher Scientific Inc., Shanghai, China). Owing to the difficulty of determining the quantity of isolated IECs, it was necessary to standardize the input of cells (about 125 μg) into each well [32]. The primary IECs were cultivated at 37 °C, 5% CO_2_ atmosphere. The culture medium was changed every other day. Morphological features of the primary cultures were inspected daily using an inverted phase-contrast microscope (Nikon TS100, Nikon Corporation, Tokyo, Japan).

### 2.3. Characterization of IEC Primary Culture

The epithelial origin of the primary cells culture was confirmed by various methods. Inverted phase-contrast microscopy was conducted before immunofluorescence (IF) staining. Through this method, cells were assessed for the presence of cobblestone morphology, crypts, and the degree of fibroblast contamination. Cytokeratin-18 (an intermediate filament specific for epithelial cells) and vimentin (an intermediate filament specific for mesenchymal cells such as intestinal fibroblast) were visualized by IF staining [33]. Cells were fixed with ice-cold methanol for 15 min, subsequently washed with PBST (PBS plus 0.05% Tween 20), and blocked for 45 min with 5% skim milk powder at room temperature (RT). The mouse anti-chicken cytokeratin 18 antibodies (Cat. MA1-06326, dilution: 1:200) diluted in PBS containing 0.5% bovine serum albumin (BSA) were added and incubated for 1 h at RT. Subsequently, cells were washed with PBST and incubated with secondary FITC-conjugated goat anti-mouse IgG antibodies. Afterward, the cells were incubated with the mouse anti-chicken vimentin antibodies (Cat. 188-10196-1, dilution: 1:400) diluted in PBS containing 0.5% BSA for 1 h at RT. Subsequently, cells were washed with PBST and incubated with secondary goat anti-mouse IgG antibodies conjugated to fluorescein Cy3 for 1 h at RT. Nuclei were stained with Hoechst 33,342 (Molecular Probes) for 10 min at RT. The stained cells were mounted using 90% glycerine in PBS with 2.5% 1,4-diazabicyclo[2.2.2]octane (Santa Cruz Biotechnology, Inc., Dallas, TX, USA), and then were imaged using an inverted epi-fluorescence microscope (Nikon Eclipse TE300; Nikon Corporation, Tokyo, Japan).

### 2.4. Time- and Dose-Dependent Mn Uptake

After the IECs grew as a monolayer in the six-well dishes (4 days post-seeding), the medium was aspirated and the cells were gently rinsed three times with uptake buffer (37 °C) comprised of 137 mmol/L choline chloride, 10 mmol/L HEPES/Tris buffer (pH 7.4), 4.7 mmol/L KCl, 1.2 mmol/L MgSO_4_, 1.2 mmol/L KH_2_PO_4_, and 2.5 mol/L CaCl_2_. For time-dependent Mn uptake assay, the uptake was initiated by adding 2 mL this uptake buffer containing 0.25 mM Mn from MnSO_4_. Uptake of Mn was arrested by discarding the uptake buffer at 0, 15, 15, 30, 45, 60, 120, 180, or 240 min post-treatment [33] and washing cells three times with ice-cold uptake buffer. For dose-dependent Mn uptake assay, the uptake was initiated by adding 1 mL uptake buffer containing 0, 0.125, 0.25, 0.50, 0.75, or 1.00 mM Mn from MnSO_4_, and Mn uptake was determined during the linear uptake period (60 min post-treatment). At each time point, cells were harvested and stored at −20 °C for Mn and protein concentrations assays.

### 2.5. Uptake of Mn from Different Mn Sources

Experiment (Expt.) 1 was conducted to investigate the influences of Fe addition on uptake of Mn from MnSO_4_ or MnLys. On day 4 post-seeding, the primary IEC from one batch of chicken embryos were randomly allotted to four treatments involving a 2 × 2 factorial arrangement. The IECs culture medium was aspirated, and the cells were gently rinsed three times with uptake buffer (37 °C). The composition of this uptake buffer is shown in the above time- and dose-dependent Mn uptake part. And then, the 2 mL of uptake buffer containing 0.25 mM Mn from MnSO_4_ or MnLys, and 0 (control) or 1.50 mM Fe from ferric ammonium citrate. The Expt. 2 and 3 were conducted to investigate the influences of NEM (a general system y^+^/CATs inhibitor) [34,35] and CHX (the inhibitor of protein synthesis) [36,37] on the uptake of Mn from MnLys respectively. On day 5 post-seeding, the IECs culture medium was aspirated, and the confluent cells were gently rinsed three times with uptake buffer (37 °C). And then, the cells were pretreated with 0 (control) or 5 μM NEM in Expt. 2, and 0 (control) or 10 μM CHX in Expt. 3 for 10 min to change the expression or modification of membrane transporters at the beginning of different Mn source addition [38,39]. After NEM or CHX pretreatment, the cells culture medium was aspirated, and the uptake buffer containing 0.25 mM Mn from MnSO_4_ or MnLys was added in the presence of NEM (Expt. 2) or CHX (Expt. 3). For all three experiments, at 60 min after Mn addition, the culture medium (2 mL) was aspirated, and the cells were washed 3 times with 2 mL ice-cold uptake buffer containing 0.05% sodium azide for 5 min to remove absorbent Mn. A part of the cells were harvested and stored at −70 °C until quantitative gene expression analyses. The remaining cells were harvested to determine Mn and protein concentrations.

### 2.6. Mn and Protein Concentrations Determination

The concentrations of Mn in the cells were measured with graphite furnace atomic absorption spectroscopy (PerkinElmer AA800; PerkinElmer Inc., Norwalk, CT, USA). The harvested cells were added to the ultrapure HNO_3_ (1:9, vol: vol), and digested using a microwave digestion unit (MARS-5; CEM Corporation, Matthew, NC, USA). The microwave procedure was performed for 45 min at 2000 W and 180 °C, and mixtures were then cooled by air to RT. Approximately 1.5 mL of digested lysate was brought to 3 mL total volume with 2% nitric acid and analyzed for Mn concentration. The limit of detection (LOD) and limit of quantification (LOQ) were 0.02 μg/L and 0.07μg/L for Mn, respectively. Bovine liver (NBS Standard Reference Material, USDC, Washington, DC, USA) was digested in ultrapure HNO_3_ and used as an internal standard for analysis (5 µg Mn/L). Protein concentration was quantified with the Bradford reagent (Sigma-Aldrich, MO, USA) using an Infinite M200Pro multi-mode plate reader and Magellan software (Tecan, CA, USA). The Mn concentrations in primary IEC are expressed as milligram Mn per gram of cell protein.

### 2.7. Quantitative Real-Time RT-PCR

The mRNA abundance for different target genes was quantified using real-time PCR [4]. Briefly, total RNA from chicken IEC was isolated using Trizol reagent (Invitrogen, Carlsbad, CA, USA) according to the manufacturer’s instructions. The integrity of the total RNA was evaluated by agarose gel electrophoresis, and the concentration was determined with a spectrophotometer (Nanodrop 2000; Thermo Fisher Scientific, Waltham, MA, USA). First-strand complementary DNA was reverse transcribed from 200 ng of total RNA using the PrimeScript^TM^ RT Reagent Kit (TaKaRa, Dalian, China). The primers for DMT1, FPN1, cationic amino acid transporter (CAT) 1, CAT2, rBAT, b^0,+^AT, and β-actin were shown in Table 1. Quantitative real-time PCR was performed in triplicate for each sample on an ABI 7500 instrument (Applied Biosystems, Foster, CA, USA). Amplification was conducted with denaturation for 15 min at 95 °C, followed by 40 cycles of denaturation for 5 s 95 °C, and annealing/elongation for 30 s at 60 °C and a final melting curve analysis. The standard curve method was used to quantify gene expression [34]. The mRNA abundance of the target gene was normalized to β-actin [4], and the relative mRNA level of the target gene in the cells treated with 0.25 mM Mn from MnSO_4_ in Expt. 1 to 3 was used as a calibrator.

### 2.8. Statistical Analysis

Analysis of the data in the time- or dose-dependent Mn uptake trial was performed by one-way ANOVA using the general linear models (GLM) procedure of SAS (SAS Institute, Inc., Cary, NC, USA). The data in Expt. 1 to 3 was analyzed by two-way ANOVA using the GLM procedure of SAS. The model included the Mn source, Fe level, and their interaction in Expt. 1. In Expt. 2 and Expt. 3, the model included Mn source, inhibitor/activator’s effect, and their interaction. The replicate well from one batch of chickens was served as the experimental unit. All data were expressed as means ± SEM (*n* = 8). When significant, post hoc comparisons of treatment means were made using Tukey’s test. Statistical significance was detected at *p* < 0.05.

## 3. Results

### 3.1. Isolation of IEC and Characterization of Primary IEC Cultures

**Figure 1:**
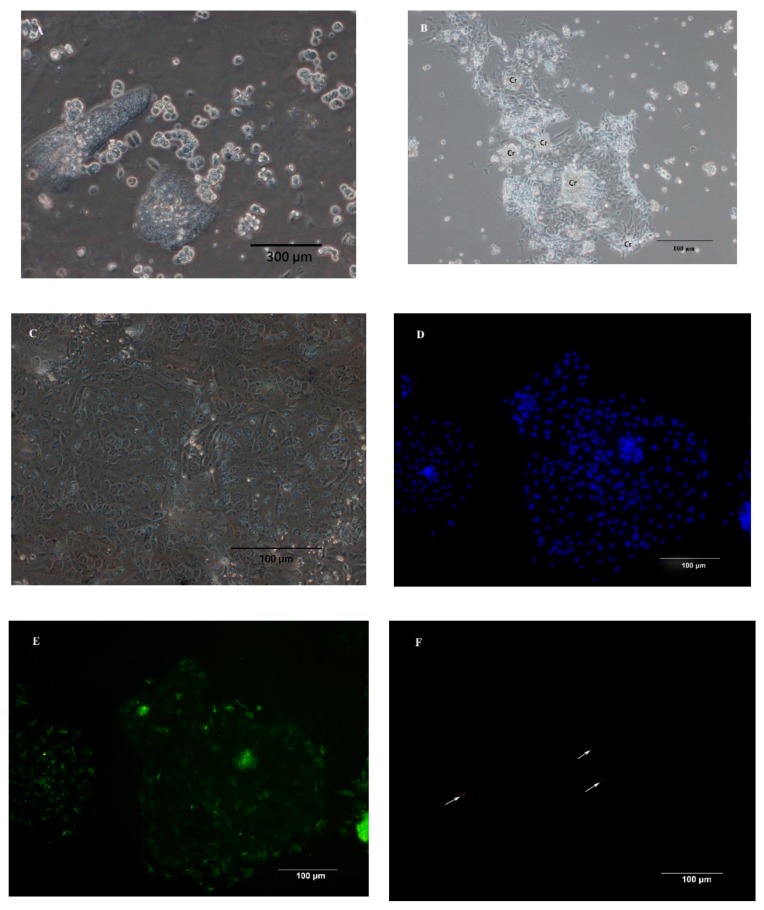

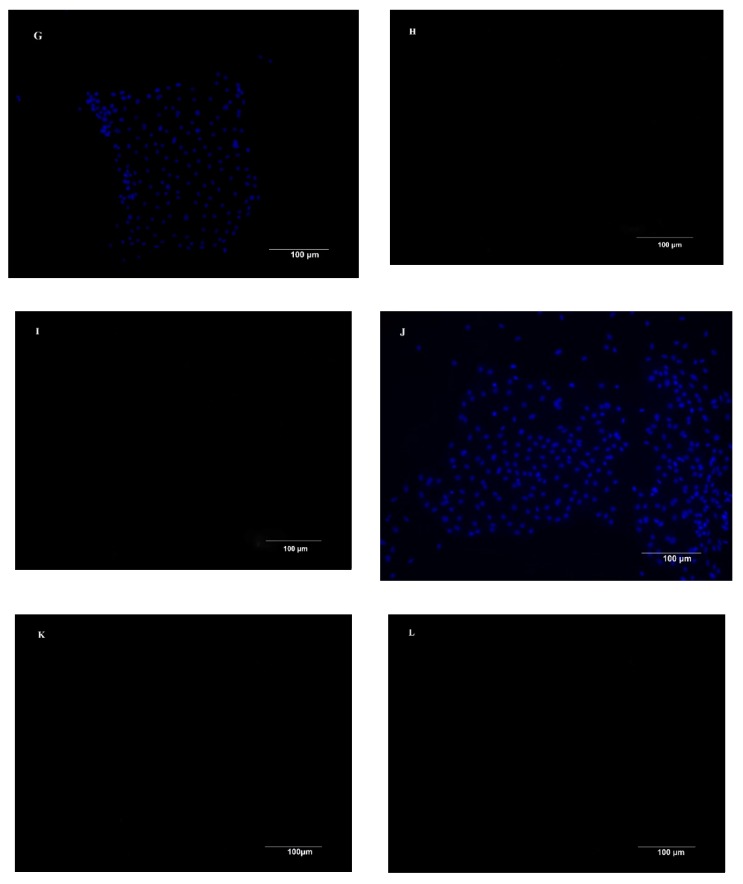
Morphological features and immunofluorescence staining of primary chicken intestinal epithelial cells (IECs). (**A**) Epithelial cells were isolated in cell clusters. (**B**) Primary chicken IECs at 24 h post-seeding. The image shows cobblestone-shaped IECs radially growing from the center of the crypt (Cr) and cell clusters connecting. (**C**) Primary chicken IECs culture within 4–5 days post-seeding. Image shows a confluent chicken IECs. (**D**,**G**,**J**) Nuclei were stained (blue). (**E**) Immunostaining against cytokeratin-18 (green) at 3 days post-seeding. (**F**) Immunostaining against vimentin (red, the arrows in the figure) at 3 days post-seeding. (**H**) Primary chicken IECs presented in the image (**G**) were incubated only with the primary antibody of cytokeratin without the secondary antibody to serve as a primary antibody control. (**I**) Primary chicken IECs presented in the image (**G**) were incubated first with buffer only without the primary antibody of cytokeratin-18 and subsequently with secondary antibody to serve as a secondary antibody control. (**K**) Primary chicken IECs presented in the image (**J**) were incubated only with the primary antibody of cytokeratin without the secondary antibody to serve as a primary antibody control. (**L**) Primary chicken IECs presented in the image (**J**) were incubated first with buffer only without the primary antibody of cytokeratin-18 and subsequently with secondary antibody to serve as a secondary antibody control.

Using a combination of dispase and collagenase, chicken duodenal IEC were isolated from the underlying basement membrane in clusters (Figure 1A). Cell adherence and attachment were 24 h after seeding, and the attached crypts consisted of a dense cell center with cells growing out of the center of the crypt in a circular manner (Figure 1B). The primary chicken IECs grew as a monolayer from the explants and exhibited an epithelial morphology with polygonal shape and cobblestone appearance at 3 to 4 days post-seeding. As shown by higher magnification, the monolayer had a typical epithelium-type mosaic structure (Figure 1C). To further characterize the phenotype of the cultured IECs isolated from the duodenum of chicken embryo, immunofluorescence staining against cytokeratin-18 (intermediate filaments typically expressed in IEC) (Figure 1E) and vimentin (intermediate filaments expressed in fibroblasts) (Figure 1F) showed that the majority of the cells (>95%) in the cultures were still of intestinal epithelial origin. No fluorescence staining was observed in the primary and second antibody controls for the cytokeratin-18 (Figure 1H,I) and vimentin (Figure 1K,L). The isolated cells proliferated 6 days on average, then underwent senescence, and stayed attached until 7 to 10 days post-seeding.

### 3.2. Time- and Dose-Dependent Mn Uptake

**Figure 2:**
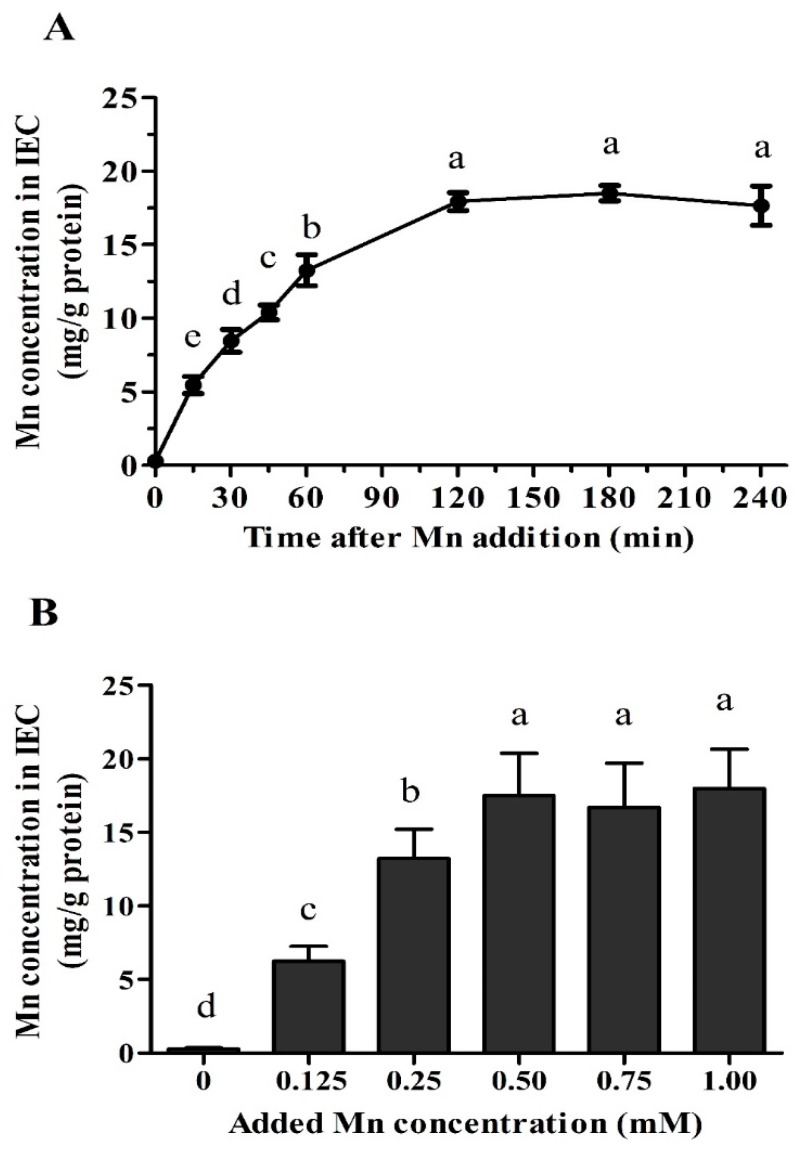
Time- and dose-dependent of Mn uptake in the primary chicken intestinal epithelial cells (IEC). (**A**) Change of Mn uptake with the time in the cells treated with 0.25 mM MnSO_4_. (**B**) Change of Mn uptake with the Mn concentration in the medium at 60 min post-seeding. Values are least-square means ± standard error (*n* = 8). ^a–e^ Mean values lacking a common letter are different at *p* < 0.05.

The Mn uptake in primary chicken IEC increased (*p* < 0.05) with time up to 120 min after 0.25 mM MnSO_4_ treatment and then plateaued (Figure 2A). Thus, the dose-dependent Mn uptake trial was performed at 60 min after Mn treatment during the linear uptake period. The Mn concentration in IECs linearly increased (*p* < 0.04) with increasing Mn concentration in the culture medium from 0 to 0.50 mM and then plateaued (Figure 2B). Hence, the comparison of uptake of Mn in primary chicken IECs from different Mn sources was performed at 60 min after 0.25 mM Mn treatment.

### 3.3. Uptake of Mn in Different Mn Sources

**Figure 3:**
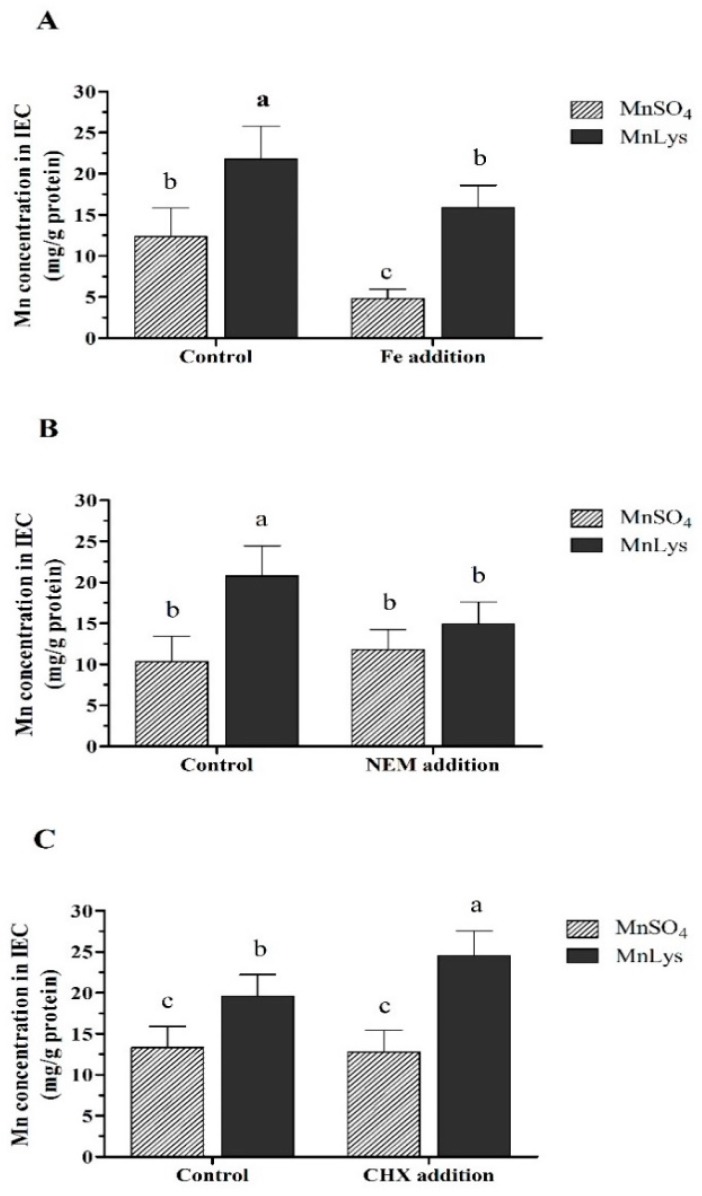
The uptake of manganese (Mn) from MnSO_4_ and Mn-lysine (MnLys) complex in the primary chicken intestinal epithelial cells (IEC). (**A**) Effect of iron (Fe) addition on the uptake of Mn from different Mn sources. The cells were treated with 0 (control) or 1.50 mM Fe (Fe addition) and 0.25 mM MnSO_4_ or MnLys for 60 min. (**B**) Influences of N-ethylmaleimide (NEM) on the uptake of Mn from different Mn sources. The cells were pretreated with 5 μM NEM for 10 min and then were treated with 0.25 mM MnSO_4_ or MnLys for 60 min in the presence of NEM. (**C**) Influences of cycloheximide (CHX) on the uptake of Mn from different Mn sources. The cells were pretreated with 5 μM CHX for 10 min and then were treated with 0.25 mM MnSO_4_ or MnLys for 60 min in the presence of CHX. Interaction of Mn source and Fe, NEM, or CHX treatment influenced (*p* < 0.05) Mn uptake in primary chicken IECs. Values are least-square means ± standard error (*n* = 8). ^a–c^ Mean values lacking a common letter are different at *p* < 0.05.

In Expt. 1, the Mn concentration in IECs was higher (*p* < 0.001) in MnLys treatment than in MnSO_4_ treatment regardless of the Fe level (Figure 3A). The Fe addition decreased (*p* < 0.05) Mn uptake when compared to the control regardless of the Mn source, while the extent of the reduction was more significant for MnSO_4_ than MnLys. In Expt. 2, the Mn uptake was increased (*p* < 0.001) in response to MnLys as compared to MnSO_4_ in primary IECs without NEM treatment (Figure 3B). The NEM treatment decreased (*p* = 0.03) the uptake of Mn from MnLys, while it did not affect (*p* = 0.42) the uptake of Mn from MnSO_4_. In Expt. 3, the Mn uptake was higher (*p* < 0.01) for MnLys than MnSO_4_ in primary IECs with or without CHX treatment (Figure 3C). The CHX treatment increased (*p* < 0.05) the uptake of Mn from MnLys but did not influence (*p* = 0.51) the uptake of Mn from MnSO_4_.

### 3.4. Transporters Expression

**Figure 4:**
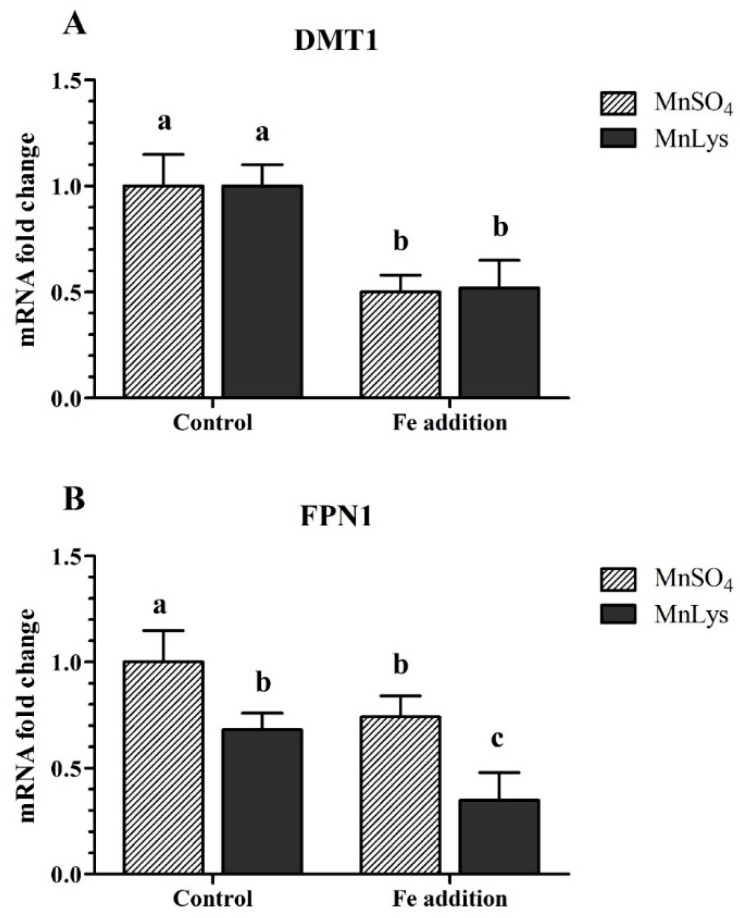
Influences of Fe addition and Mn source on ionized Mn^2+^ transporters expression in primary chicken intestinal epithelial cells (IECs). The primary confluent IECs were incubated with 0 or 1.50 mM Fe (ferric ammonium citrate), and 0.25 mM Mn from MnSO_4_ or Mn-lysine complex (MnLys) for 60 min. The mRNA abundance of divalent metal transporter 1 (DMT1) (**A**) and Figure 1. (FPN1) (**B**) were determined by real-time RT-PCR. The Fe addition affected DMT1 mRNA abundance, and Mn source and Fe addition influenced FPN1 transcriptional expression (*p* < 0.05). Values are least-square means ± standard error (*n* = 8). ^a–c^ Mean values lacking a common letter are different at *p* < 0.05.

In Expt. 1, the Fe addition decreased (*p* < 0.05) the mRNA abundance of DMT1 in primary chicken IECs, while there was no significant difference (*p* > 0.31) between MnSO_4_ and MnLys treatments (Figure 4A). The Fe addition also decreased (*p* < 0.05) the mRNA abundance of FPN1 in primary chicken IECs, and the FPN1 mRNA level was lower (*p* < 0.02) in MnLys treatment than MnSO_4_ treatment (Figure 4B).

**Figure 5:**
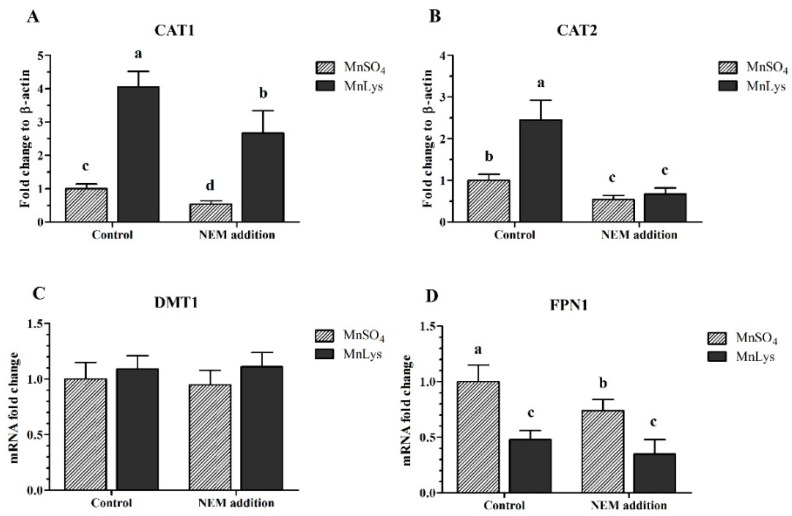
Influences of *N*-ethylmaleimide (NEM) and Mn source treatments on the membrane transporters genes expression in the primary chicken intestinal epithelial cells (IECs). The primary confluent IECs were pretreated with 5 μM NEM for 10 min and then were incubated with 0.25 mM Mn from MnSO_4_ or Mn-lysine complex (MnLys) for 60 min in the presence of NEM. The mRNA abundance of cationic amino acid transporter (CAT) 1, CAT2, divalent metal transporter 1 (DMT1), and ferroportin 1 (FPN1) was determined by real-time RT-PCR. Interaction of Mn source and NEM addition influenced CAT1, CAT2, and FPN1 transcriptional expression (*p* < 0.05). Values are least-square means ± standard error (*n* = 8). ^a–d^ Mean values lacking a common letter are different at *p* < 0.05.

In Expt. 2, the MnLys increased (*p* < 0.001) CAT1 and CAT2 mRNA expression in control without NEM treatment, while it only increased (*p* < 0.001) CAT1 mRNA expression in the NEM-treated cells when compared to MnSO_4_ (Figure 5A,B). The NEM addition decreased (*p* < 0.001) the mRNA abundance of CAT1 and CAT2 in primary chicken IECs, while the extent of the reduction was greater for MnLys than MnSO_4_. The MnLys decreased (*p* < 0.001) FPN1 mRNA expression when compared to MnSO_4_ in primary chicken IECs with or without NEM treatment (Figure 5D). The NEM addition decreased (*p* = 0.01) FPN1 mRNA abundance in MnSO_4_-treated cells, but did not influence (*p* > 0.20) FPN1 mRNA abundance in MnLys-treated cells. The Mn source, NEM treatment, and their interaction did not influence (*p* > 0.12) DMT1 mRNA expression in primary chicken IECs (Figure 5C).

**Figure 6:**
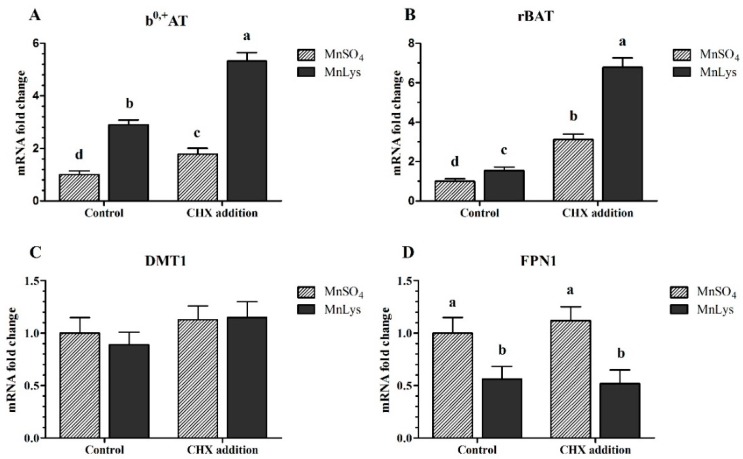
Influences of cycloheximide (CHX) and Mn source treatments on the membrane transporters genes expression in the primary chicken intestinal epithelial cells (IECs). The primary confluent IECs were pretreated with 10 μM CHX for 10 min and then were incubated with 0.25 mM Mn as MnSO_4_ or Mn-lysine complex (MnLys) for 60 min in the presence of CHX. The mRNA abundance of transport system b^0,+^ components rBAT and b^0,+^AT, and ionized Mn transporters divalent metal transporter 1 (DMT1), and ferroportin 1 (FPN1) was determined by real-time RT-PCR. Interaction of Mn source and CHX treatment influenced rBAT and b^0,+^AT expression but only the Mn source affected the FPN1 mRNA abundance (*p* < 0.05). Values are least-square means ± standard error (*n* = 8). ^a–d^ Mean values lacking a common letter are different at *p* < 0.05.

In Expt. 3, the MnLys increased (*p* < 0.001) b^0,+^AT and rBAT mRNA expression when compared to MnSO_4_ in primary chicken IECs with or without CHX treatment (Figure 6A,B). The FPN1 mRNA abundance was decreased (*p* = 0.001) in response to MnLys as compared to MnSO_4_ in primary chicken IECs. The CHX treatment increased (*p* < 0.001) the mRNA abundance of b^0,+^AT and rBAT in primary chicken IECs, and the extent of increase was greater for MnLys than MnSO_4_. However, the CHX treatment did not influence (*p* = 0.56) FPN1 mRNA expression in primary chicken IECs regardless of Mn source treatment (Figure 6D). The Mn source, NEM treatment, and their interaction did not influence (*p* > 0.12) DMT1 mRNA abundance in primary chicken IECs (Figure 6C).

## 4. Discussion

Duodenum, with the high number of crypts and villi, was selected as the starting material for the isolation of IECs [35]. In this study, we established a primary chicken IECs model from the duodenum of chicken embryos for investigating Mn uptake. Digestion of the duodenal epithelium with collagenase and dispase allowed for the isolation and cultivation of viable intact organoid, which was consistent with the previous studies [31,36]. The crypts are essential to generate proliferating IECs because they contain viable stem cells [35]. Morphologically, the attached crypts comprised a dense cell center with cells growing out of the center of the crypts circularly. And then chicken IECs proliferated to a monolayer of cuboidal cells at 3–4 days post-seeding. The morphological characteristics of chicken IECs were similar to the primary rat IECs but the time for forming confluence monolayer was earlier in primary chicken IECs than in primary rat IECs [33]. The presence of cytokeratin-18, an intermediate cytoskeletal filament that traverses the cell cytoplasm, is characteristic of IECs [37], and vimentin is an intermediate filament specific for intestinal fibroblast [40]. The immunofluorescence staining results showed that primary cells culture consisted predominantly of IEC at 2–3 days post-seeding. The high purity of primary cells supports that our primary IECs cultures are a valid model to characteristics of Mn uptake.

Graphite furnace atomic absorption spectroscopy possesses adequate sensitivity to measure intracellular Mn concentrations [41]. This method provided a viable alternative to using radiolabeled Mn in metal transport studies with sufficient sensitivity to study the uptake of Mn in a cell culture model. In this study, we found that there was higher Mn uptake from MnLys than MnSO_4_ in primary IECs without other treatments, which was consistent with the previous finding using intact chicken and ligated intestinal loops [5,42]. Much research has shown that amino acids facilitated Mn uptake in the small intestine of broilers, but the mechanisms were unknown [5,42,43]. To investigate the potential pathways of uptake of Mn from MnLys complex, we added Fe, NEM (a system y^+^/CATs inhibitor), and CHX (an inhibitor of protein synthesis) in the culture media to determine their influences on Mn uptake from MnLys and MnSO_4_.

Firstly, iron addition decreased Mn uptake in chicken IECs regardless of the Mn source. This result demonstrated that uptake of Mn from MnLys and MnSO_4_ at least shared one transport pathway that can be regulated by iron. The dissociated Mn^2+^ from Mn sulfate is transported by the cellular influx transporter DMT1 and cellular efflux transporter FPN1 in the small intestine of animals [44,45]. The decrease in DMT1 gene expression by Fe addition might have caused the decrease of Mn uptake in primary IECs. This result agreed with previous studies that Fe-sufficient condition repressed DMT1 expression [46,47,48], resulting in less Mn absorption in the gut of animals [49,50]. Furthermore, we found there was higher Mn uptake from MnLys than MnSO_4_ in primary chicken IECs with Fe treatment. Decreased FPN1 expression was associated with decreased Mn efflux in human embryonic kidney cells and human epithelial HeLa cells [16,51]. The decrease in FPN1 expression presented herein indicated that MnLys might reduce Mn efflux in primary chicken IECs as compared to MnSO_4_. The Mn accumulation in primary chicken IECs from MnLys to some extent was due to the decrease in Mn efflux. Besides, there was no significant difference in DMT1 mRNA between MnLys- and MnSO_4_-treated cells. However, Bai et al. found that supplementation with an Mn-amino acid chelate increased DMT1 mRNA in the small intestine of broilers when compared to supplementation with MnSO_4_ [4,5]. Previous studies found that Fe status also influenced DMT1 mRNA expression in cells [46,47,48]. However, we did not determine Fe concentration in primary IECs. Thus, further research should be conducted to investigate the effects of MnLys on the concentration of Fe in primary chicken IECs.

Secondly, in the MnLys molecules, the lysine binds in what is known as a glycinato manner, where a five-membered ring is formed with metal-amino nitrogen and carboxylic oxygen [52]. The results of double isotope studies using radiolabeled metal and ligand simultaneously also identified the metal-amino acid occurrence in intestinal mucosal cells transported from the gastrointestinal lumen [53,54]. The metal-amino acid chelate may be absorbed into the mucosal cell in the form of intact molecular through amino acid transporter [20,21,22]. Cannon et al. concluded that L-cystine transporters were involved in the uptake of mercuric conjugates of cysteine [55]. Their findings would imply that the same applies to other metals chelated to other amino acids. Two transport systems, b^0,+^ and y^+^, were showed to transport L-lysine across the apical membrane of enterocytes [23]; thus we hypothesized that the uptake of MnLys was into the cell through L-lysine specific transporters y^+^/CATs and b^0,+^. In this study, uptake of Mn from MnLys was decreased in primary chicken IECs response to NEM, a general system y^+^/CATs inhibitor [38,56,57], which suggested that the system y^+^/CATs plays a role in the uptake of Mn from MnLys. One explanation of the increase of Mn uptake from MnLys might be due to the increases of CAT1 and CAT2 mRNA expression in the primary chicken IECs without NEM treatment. The CHX treatment induced the uptake of L-lysine via the activation of system b^0,+^ in Caco-2 cells [30]. The increased b^0,+^AT and rBAT mRNA abundance present herein supported that CHX is an activator of system b^0,+^, as described in the Caco-2 cells model [28,29,30]. We also found that the CHX treatment increased uptake of Mn from MnLys, which indicated that the system b^0,+^ was involved in the uptake of Mn from MnLys in chicken IEC. The increase of Mn uptake from MnLys was at least partly due to the activation of system b^0,+^ in the primary chicken IECs without NEM treatment. Similarly, Gao et al. [58] demonstrated that Cu^2+^ was transported as a complex with methionine into the epithelium and that uptake was mediated by an amino acid transporter as excessive methionine inhibited the transport of the Cu^2+^. On the other hand, NEM and CHX treatment did not influence the ionized Mn^2+^ transporter DMT1 and FPN1 expression, also suggesting that the Mn^2+^ transport pathway was not involved in the changes in uptake of Mn from MnLys after NEM or CHX treatment.

In conclusion, there was greater uptake of Mn from MnLys than MnSO_4_ in primary chicken IECs. Iron decreased Mn uptake irrespective of the Mn source. The Mn accumulation might be regulated by Mn efflux, because of an alteration in basolateral FPN1 expression in primary chicken IECs. The NEM, an inhibitor of system y^+^/CATs, decreased, while the CHX, an activator of system b^0,+^, increased uptake of Mn from MnLys. The uptake of Mn from MnLys complex is likely not only mediated through transporters specific for ionized Mn^2+^, but also through CAT1/2 and system b^0,+^ amino acid transporters in primary chicken IECs.

## Figures and Tables

**Table 1:** The primers for quantitative real-time PCR.

Gene	Primer	Sequence (5′-3′)	GeneBank ID
DMT1	F	CATGTACTTCGTGGTGGCCT	EF635923
	R	GATCAGACACAGCCACGTCA	
FPN1	F	GATGCATTCTGAACAACCAAGGA	GI 61098365
	R	GGAGACTGGGTGGACAAGAACTC	
CAT1	F	CAAGAGGAAAACTCCAGTAATTGCA	XM_417116
	R	AAGTCGAAGAGGAAGGCCATAA	
CAT2	F	TGCTCGCGTTCCCAAGA	XM_420685
	R	GGCCCACAGTTCACCAACAG	
b^0,+^AT	F	CAGTAGTGAATTCTCTGAGTGTGAAGCT	XM_414130
	R	GCAATGATTGCCACAACTACCA	
rBAT	F	CCCGCCGTTCAACAAGAG	XM_426125
	R	AATTAAATCCATCGACTCCTTTGC	
β-actin	F	FGAGAAATTGTGCGTGACATCA	L08165
	R	CCTGAACCTCTCATTGCCA	

*DMT1*, divalent metal transporter 1; *FPN1*, ferroportin 1; *CAT*, cationic amino acid transporter.
